# Coordination and equilibrium selection in games: the role of local effects

**DOI:** 10.1038/s41598-022-07195-3

**Published:** 2022-03-01

**Authors:** Tomasz Raducha, Maxi San Miguel

**Affiliations:** 1 Instituto de Física Interdisciplinar y Sistemas Complejos IFISC (CSIC-UIB), Palma, Spain; 2 Institute of Experimental Physics, Faculty of Physics, University of Warsaw, Warsaw, Poland

**Keywords:** Complexity, Computer modelling, Emergence, Population dynamics, Applied physics, Phase transitions and critical phenomena, Complex networks, Phase transitions and critical phenomena, Applied mathematics, Environmental economics, Population dynamics

## Abstract

We study the role of local effects and finite size effects in reaching coordination and in equilibrium selection in two-player coordination games. We investigate three update rules — the replicator dynamics (RD), the best response (BR), and the unconditional imitation (UI). For the pure coordination game with two equivalent strategies we find a transition from a disordered state to coordination for a critical value of connectivity. The transition is system-size-independent for the BR and RD update rules. For the IU it is system-size-dependent, but coordination can always be reached below the connectivity of a complete graph. We also consider the general coordination game which covers a range of games, such as the stag hunt. For these games there is a payoff-dominant strategy and a risk-dominant strategy with associated states of equilibrium coordination. We analyse equilibrium selection analytically and numerically. For the RD and BR update rules mean-field predictions agree with simulations and the risk-dominant strategy is evolutionary favoured independently of local effects. When players use the unconditional imitation, however, we observe coordination in the payoff-dominant strategy. Surprisingly, the selection of pay-off dominant equilibrium only occurs below a critical value of the network connectivity and disappears in complete graphs. As we show, it is a combination of local effects and update rule that allows for coordination on the payoff-dominant strategy.

## Introduction

Voluntary individual efforts are often crucial for the collective survival. Cooperation might be equally important in an economic system, a small social group, or a transportation network, increasing efficiency of personal actions. In many man-made systems proper regulations enforce that we end up in an optimal configuration, i.e. that we coordinate. However, in some cases global supervision is impossible and local willingness to cooperate does not ensure coordination. Nevertheless, in many complex systems coordination emerges without an external control or regulation. Bottom-up initiatives shaping our world regularly arise without any supervision, driven purely by local interactions. Understanding this phenomenon is of a paramount importance in the context of global challenges that require coordinated efforts, like pandemics^1,2^ and climate change^3,4^, but also in explaining our evolution and in the emergence of language^5–7^.

The increase in size and complexity of social structures gives rise to the question of how to organise them in harmony. Any solution to this problem requires an understanding of which local processes drive human coordination and how they can be used to reinforce the unity of action. These questions are often studied by analysing so-called coordination games^8–11^. In such framework, two or more agents are involved in a game whose outcome is more profitable for everyone if they choose to play the same strategy. This approach helps explaining outcomes of coordination dilemmas. The question of equilibrium selection refers to the underlying trade off between benefit (payoff dominance) and safety (risk dominance) which is the essence of many organisational and economic challenges.

Our approach is based on evolutionary game theory^12–15^. A population of players can interact through a network of connections. An interaction consists of playing a game specified by a payoff matrix. The strategy played by individuals evolves over time, as they adapt to the strategies played by their neighbours and their own payoff or fitness. The players always try to increase their payoffs, or in other interpretation those with higher fitness spawn more replicas of themselves. Evolutionary game theory has applications in a range of fields, from economics to sociology to biology^16–18^. Usually, in theoretical or computational analysis, an arbitrary procedure of updating the strategy of individuals during the evolutionary game is considered. Here, we also explore the impact of the selected update rule on emergence of coordination among agents in different games. Local effects, however, can have a crucial influence on the coordination. We test a wide range of connectivity and system sizes to see which features have the largest impact on the collective behaviour.

We study equilibrium selection under different update rules by investigating a spectrum of coordination games played in a population of agents. We look at their behaviour in a network of *N* nodes, each with a degree *k*, i.e. connected to other *k* agents in the network. We consider a fixed structure of networks only, temporal networks^19,20^ and coevolving networks^21,22^ are out of the scope of this article. Our numerical simulations are performed for random regular graphs, and Erdős–Rényi networks where specified^23^. We use the *igraph* python package^24,25^ to construct the networks (more specifically $\texttt {K\_Regular}$ and $\texttt {Erdos\_Renyi}$ functions). We analyse two-player games, i.e. only two players can be involved in the game at a time. Such games are described by a $2 \times 2$ payoff matrix:

1 $$\begin{gathered} \begin{array}{*{20}c} {} & {\quad \; {\text{A}}} &\quad {\text{B}} \\ \end{array}\hfill \\ \begin{array}{*{20}c} {\text{A}} \\ {\text{B}} \\ \end{array} \left( {\begin{array}{*{20}c} R & S \\ T & P \\ \end{array} } \right) \hfill \\ \end{gathered}$$

where A and B are two strategies available to the players (we only consider pure strategies). Parameters *R*, *S*, *T*, and *P* define the payoffs of the row player and do not change during the evolution of the system. Note, that coordination games are defined by $R>T$ and $P>S$. A special case of a coordination game is the stag hunt game for which $R>T>P>S$^26^. In many cases we can distinguish a payoff-dominant (aka Pareto-optimal) and a risk-dominant strategy^27^. A payoff-dominant strategy is the one which gives the largest absolute payoff. A risk-dominant strategy is the one which gives the largest expected payoff, assuming that the opponent will play either strategy with equal probabilities. This means that the risk-dominant strategy is the safest option in lack of information. For the payoff matrix (1) the strategy A is payoff-dominant if $R>P$, and the strategy B is risk-dominant if $R-T<P-S$. When all players coordinate on one of these strategies we refer to such state as a payoff-dominant or risk-dominant equilibrium.

We run numerical simulations with agents initially using a random strategy. At the beginning of each simulation every node is assigned a strategy A or B with equal probability. Then, before starting with the actual algorithm each agent plays the game with every neighbour and receives the corresponding payoff. This procedure sets up the initial payoff for the simulation. Note, that assigning any fixed initial payoff (like zero) to every node instead of establishing it based on the initial strategies can lead to considerably different results. To analyse the time evolution of the system, we update the state of nodes, i.e. the last payoff and the used strategy, in each time step until a stationary state or a frozen configuration is reached. We use random asynchronous update which prevents formation of trapped oscillating states. In every time step a node, called a focal or active node, is randomly selected to play the game with its neighbours and update its strategy based on its payoff and the applied update rule. There are several update rules used in the literature on evolutionary game theory^28,29^. We use three of the most common ones:

- the Replicator Dynamics (RD) (aka replicator rule, or proportional imitation rule)—the active node compares the payoff with a random neighbour and copies its strategy with probability $p=(\mathrm {payoff~diff.})/\phi$, if the neighbour’s payoff is bigger. Normalisation $\phi$ is the largest possible payoff difference allowed by the payoff matrix and network structure and it sets the probability *p* within [0, 1] range,
- the myopic Best Response (BR)—the active node chooses the best strategy given the current strategies of the neighbours, i.e. it compares all payoffs it would obtain playing each possible strategy against the current strategies of the neighbours and chooses the strategy resulting in the largest payoff,
- the Unconditional Imitation (UI)—the active node copies the strategy of the most successful neighbour, i.e. the one with the highest payoff, if its payoff is larger.

Another relatively popular rule is the Fermi update rule^30,31^. It is similar, however, to the replicator dynamics in nature, but introduces an additional parameter accounting for noise or temperature. The replicator dynamics can be seen mostly in biological applications^18,32,33^, whether to describe replication of genes, species, or individuals. The best response update rule is usually considered in the economics literature^34–39^, where the assumption of rational agents is typical. The unconditional imitation is popular in complexity and social science^14,40–44^, as it resembles social imitation. Surprisingly, all three update rules can lead to substantially different outcomes, therefore the evolutionary game environment is defined by the payoff matrix as much as by the update rule^45–50^. Consequently, it is crucial to understand the implications of each update rule in order to be able to compare it to empirical results and identify rules that are actually used by humans. Both experimental and theoretical work are necessary to face this challenge. While many experiments are being performed to uncover the mechanisms behind human decision making when playing games^51–55^, we address here the theoretical implications of different updating mechanisms.

We focus on the role of local effects and finite size effects on reaching coordination and equilibrium selection under different update rules. A number of previous results, described later in detail, suggest that local effects are more important for update rules which have an imitative nature, such as unconditional imitation^45,46,56^. A particularly important specific result^56^ establishes that for sparse networks, above a threshold connectivity of agents placed on a circle, and using unconditional imitation the Pareto-optimal equilibrium is selected, and below this threshold the risk-dominant equilibrium is selected. We find here a different behaviour in random networks—the Pareto-optimal equilibrium is only selected below a threshold connectivity. In very sparse networks we do not obtain full coordination. This highlights the need of a quantitative and detailed study of local effects in equilibrium selection.

In order to measure the level of coordination we incorporate a parameter $\alpha \in [0,1]$, called coordination rate, accounting for the fraction of nodes using the strategy A. Therefore, $\alpha = 1$ indicates a full coordination in the system with every agent using the strategy A, while $\alpha = 0$ indicates a full coordination on the strategy B. To characterise the evolution of the system we additionally use the fraction of active connections $\rho \in [0,1]$ (interface density) and the convergence time $\tau$. An active connection is a link between two nodes in different states, i.e. using different strategies. The convergence time is the time at which the system achieves a frozen configuration, always counted in Monte Carlo (MC) time steps. If instead of the frozen configuration the system stabilises in an active stationary state, the convergence time $\tau$ will reach the maximal limit indicated in individual cases.

## Previous results in coordination games

Coordination games have been extensively studied in the past. In particular, in two-player games the competition between a payoff-dominant and risk-dominant equilibrium gained a lot of attention. When the payoff matrix is constructed in such a way that one strategy is payoff-dominant and the other is risk-dominant, it is in principle not clear which strategy will be favoured by the population. Therefore, a number of authors have investigated how a stochastically stable equilibria is chosen and approached by the system^57^.

Most of the work has been focused on the myopic best response update rule, with some interest in imitation rules as well^8^. The KMR model explored the equilibrium selection in well mixed populations with players updating their strategies according to the best response rule^34^. The model is an equivalent of interactions on a complete or fully connected graph. The unique long run equilibrium was found to correspond to every agent playing the risk-dominant strategy. In other words, the risk minimisation was more important than the profit maximisation for players. The model was extended to include n-player games, different sub-populations and asymmetric interactions with similar results^35,58–60^ (note that the concept of risk-dominance can be generalised for n-player games). In the KMR model the focal player, when deciding about its strategy update, compares payoffs from two strategies in the current state of the population. As later noticed, this process is in reality of imitative nature, since effectively the player will use the strategy that gained the most in the last round. The model was extended to include in the payoff computation the fact that the focal node will change it’s state and therefore influence the future state of the system, but no differences were detected^38^. Note, that for large networks the effect of this extension of the KMR model is negligible.

A well-mixed population is the first approximation of how social structures can look like. In reality they are much more complex^61–63^. Note, that in the approximation of a well-mixed system the best response and unconditional imitation will give the same results. First extensions of the KMR model onto different topologies included a *circular city*, i.e. players placed in a circle interacting only with a fixed number *k* of nearest neighbours^37^. This version solved the problem of very slow convergence, which could undermine the idea that the long run equilibrium can be ever obtained in the real world. However, the basic outcome was the same—coordination in the risk-dominant strategy was preferred over coordination in the payoff-dominant one. This work was performed still assuming the myopic best response. When using unconditional imitation in the circular city model, coordination in the payoff-dominant strategy could be found^56^. Additionally, equilibrium selection in such case depends on the degree *k* of the network in a non-monotonic manner. There is a threshold value $k^*$: if players are connected to more neighbours ($k>k^*$) the Pareto-optimal equilibrium can be achieved. An additional requirement for the minimal network size is imposed—obviously for *k* approaching *N* we should expect results obtained before for complete graphs and those favour risk-dominant equilibrium. Therefore, in the circular city model^56^ when increasing the degree from $k=2$ to a complete graph we should observe risk-dominance, then payoff-dominance, and finally risk-dominance again. This was the first clear evidence that equilibrium selection can depend on the connectivity of the network (apart from the update rule). Note, that we report a different effect of the connectivity in our simulations of random graphs where no coordination is achieved for very sparse networks. However, we do not allow for random mutations. Other attempts to go beyond a fully connected population included random matching mixed with an imitative update rule^64^. Such an environment favoured the payoff-dominant equilibrium as well. However, in network science this would be an equivalent of a very particular temporal network with random regular structure of degree $k=1$. A recent work considered the mentioned before circular city model, but with a random and changing in time interaction structure^65^. The update rule was also unconditional imitation and the simulation always included random mutations. The structure of the network, however, was changing allowing the agents to play the game with a random, though limited, subset of other agents. Additionally, the structure of interactions was different than the structure of information network. Agents would use a different group of players to interact and to compare the payoffs, although both groups could overlap. In this specific setup more interactions lead to a lower probability of obtaining the payoff-dominant equilibrium. In other words, the circular city model with a higher temporal degree will more likely favour the risk-dominant equilibrium. This result is qualitatively consistent with our results for the general coordination game on non-sparse networks, although we investigate different topologies.

The work discussed above assumed synchronous update which can lead to traps of the dynamics including oscillatory states and strange symmetric patterns, like in the prisoners dilemma on lattice^14^. For this reason those models allow for noise in a form of random mutations (errors in applying the update rule) and theoretical results are computed in the limit of the mutation rate going to zero. Another way of avoiding the traps of synchronous update is using the asynchronous update, as we do in this paper, which is stochastic by nature.

Equilibrium selection in coordination games has been later studied on more complex networks and with various additional features imitating real social interactions. High clustering of the network proved to be important in equilibrium selection for models with mutations and best response^66^. Other computational study for myopic best response showed that the payoff-dominant equilibrium may be chosen more frequently if the average degree of the network is larger and the network is more centralised, i.e. the maximum degree is bigger^67^. This last study was performed, however, for very small network sizes and other works didn’t confirm the importance of centrality^66^ or reported inhibited cooperation in the stag hunt game under unconditional imitation^46^. The case of random regular graphs has been investigated theoretically in the limit of infinite networks together with the limit of weak selection, i.e. with an update rule asymptotically independent of the payoff^45^. The results showed that imitative update rules in cooperation games, with probability of imitation proportional to fitness, can favour Pareto-efficiency over risk dominance. The same tendency was shown for unconditional imitation in random networks with small degree for the Stag–Hunt game^46^.

In time dependent networks, which we do not consider in this paper, the possibility of changing costly links has been explored and showed that mixed equilibria that are not risk-dominant nor Pareto-optimal are possible^68^. Frozen disordered configurations were characterised in a coevolutionary model where the network also evolves and demonstrated to be stochastically stable^69^. Coevolution of node strategy and network topology in evolutionary game theory has been reviewed elsewhere^22^. The idea of separating the group in which we interact (i.e. play the game) from the one where we learn (i.e. update our strategy) was also considered^65,70–72^. In the case of interacting only locally, but observing and imitating beyond the nearest neighbourhood, a condition was provided to observe the payoff-dominant equilibrium^73^. Another extension considered mixed populations with agents using different update rules^74^, indicating that the mixing can be destructive for coordination. Those considerations, however, go beyond the scope of this paper. We consider exclusively fixed networks with no coevolution or time-dependence, and pure strategies with a single update rule used at a time.

Although equilibrium selection has been extensively studied in the past there are still many open questions. In general, coordination games were mostly studied under myopic best response or on a complete graph. The Stag Hunt game is an exception, having been studied with several update rules and for more complex structures^46,66^. However, other possible configurations of payoff matrices were investigated only theoretically with idealised assumptions, which often are not feasible in social systems. A precise dependence of equilibrium selection on the network’s degree is also missing. Numerical work was performed for very sparse networks, while theoretical predictions were calculated for complete graphs or regular structures like lattices and rings. Strict comparison of coordination games on complex networks under RD, BR, and UI update rules is also lacking. Those gaps are filled by our work.

Much less effort has been put in the study of pure coordination games, i.e. games in which the pay-off matrix is such that coordination in strategy A or B are two equivalent equilibria. It was shown that the average frequency found for each strategy is 1/2, as expected^66^. It is, however, unclear when this value comes from full coordination in each of the strategies in half of the realisations of the statistical sampling, and when the system ends up in a non-coordinated state with half of the players using one strategy and the other half the other strategy. This question is especially important since it was predicted that players using the BR update rule on regular structures may end up in a disordered frozen configuration, i.e. without reaching global coordination^75^. We explore the problem of coordination with two equivalent equilibria in the following section.

## Results

### Pure coordination game

In this section we study the Pure Coordination Game (PCG) (also known as doorway game, or driving game) in which $R=1$, $S=0$, $T=0$, and $P=1$, resulting in a symmetric payoff matrix with respect to the two strategies:

2 $$\begin{gathered} \begin{array}{*{20}c} {} & {\quad \; {\text{A}}} &\; {\text{B}} \\ \end{array}\hfill \\ \begin{array}{*{20}c} {\text{A}} \\ {\text{B}} \\ \end{array} \left( {\begin{array}{*{20}c} 1 & 0 \\ 0 & 1 \\ \end{array} } \right) \hfill \\ \end{gathered}$$

There are two equivalent equilibria for both players coordinating at the strategy A or B (a third Nash equilibrium exists for players using a mix strategy of 50% A and 50% B). As the absolute values of the payoff matrix are irrelevant and the dynamics is defined by ratios between payoffs from different strategies, the payoff matrix (2) represents all games for which the relation $R=P>S=T$ is fulfilled.

In the PCG the dilemma of choosing between safety and benefit does not exist, because there is no distinction between risk-dominant and payoff-dominant equilibrium. Both strategies yield equal payoffs when players coordinate on them and both have the same punishment (no payoff) when players fail to coordinate. Therefore, the PCG is the simplest framework to test when coordination is possible and which factors influence it and how. It is in every player’s interest to use the same strategy as others. Two strategies, however, are present in the system at the beginning of the simulation in equal amounts. From the symmetry of the game we can expect no difference in frequency of each strategy being played, when averaged over many realisations. Still, the problem of when the system reaches full coordination in one of the strategies is not trivial. We address this question here.

**Figure 1:**
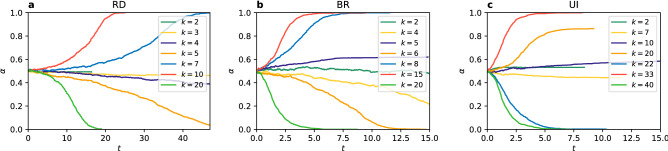
Time evolution of the coordination rate $\alpha$ (in MC steps) in individual realisations for different values of the degree *k* in a random regular network of $N=1000$ nodes, using (**a**) the replicator dynamics, (**b**) the best response, and (**c**) the unconditional imitation update rule.

**Figure 2:**
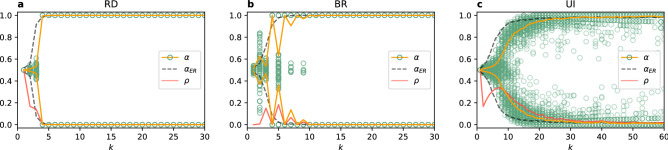
Coordination rate $\alpha$ and interface density $\rho$ vs degree *k* of a random regular network for $N=1000$ using (**a**) the replicator dynamics, (**b**) the best response, and (**c**) the unconditional imitation update rule. Each green circle represents one of 500 realisations for each value of the degree *k* and the average value is plotted with a solid line, separately for $\alpha >0.5$ and $\alpha \le 0.5$. Results are compared to the ER random network ($\alpha _{ER}$) with the same average degree.

First, we look at single trajectories as presented in Fig. 1. Some of them quickly reach $\alpha =0$ or 1, or stop in a frozen state without obtaining global coordination. Other trajectories take much longer and extend beyond the time scale showed in the figure. What we can already tell is that the process of reaching coordination is slower in the replicator dynamics where it usually takes more time than in the best response and unconditional imitation to reach a frozen configuration. For all update rules the qualitative effect of the connectivity is similar—for bigger degree it is more likely to obtain full coordination and it happens faster. For the UI, however, larger values of degree than for the RD and BR are required to observe coordination. For example, in the case of $k=10$ or 20 the system stops in a frozen disorder when using UI, while for the RD and BR it quickly reaches a coordinated state of $\alpha =0$ or 1.

To confirm the conclusions from observation of trajectories, we present the average outcome of the system’s evolution in the Fig. 2. The first thing to notice is that all plots are symmetrical with respect to the horizontal line of $\alpha = 0.5$. It indicates that the strategies are indeed equivalent as expected. In all cases there is a minimal connectivity required to obtain global coordination. For the RD and BR update rules this minimum value is $k=4$, although in the case of BR the system fails to coordinate for small odd values of *k* due to regular character of the graph. This oscillating behaviour does not exist in Erdős–Rényi random networks. When nodes choose their strategies following the UI rule much larger values of *k* are required to obtain full coordination. Single realisations can result in $\alpha = 0$, or 1 already for $k=15$. However, even for $k=60$ there is still a possibility of reaching a frozen uncoordinated configuration.

The important conclusion is that there is no coordination without a sufficient level of connectivity. In order to confirm that this is not a mere artefact of the random regular graphs we compare our results with those obtained for Erdős–Rényi (ER) random networks^76,77^ (black dashed line in Fig. 2). The level of coordination starts to increase earlier for the three update rules, but the general trend is the same. The only qualitative difference can be found in the BR. The oscillating level of coordination disappears and it doesn’t matter if the degree is odd or even. This shows that different behaviour for odd values of *k* is due to topological traps in random regular graphs^78^. Our results for the UI update rule are also consistent with previous work reporting coordination for a complete graph but failure of global coordination in sparse networks^40^.

**Figure 3:**
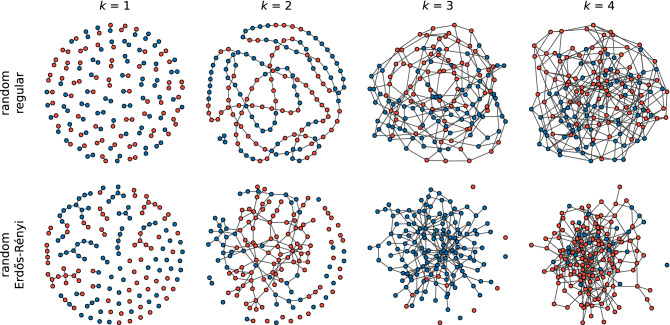
Examples of frozen configuration reached under the UI update rule for small values of the average degree *k* in random regular networks (top row) and Erdős–Rényi networks (bottom row) with 150 nodes. Red colour indicates a player choosing the strategy A, blue colour the strategy B. Note the topological differences between random regular and ER networks when they are sparse. For $k=1$ a random regular graph consists of pairs of connected nodes, while an ER network has some slightly larger components and many loose nodes. For $k=2$ a random regular graph is a chain (sometimes 2–4 separate chains), while an ER network has one large component and many disconnected nodes. For $k=3$ and $k=4$ a random regular graph is always composed of one component, while an ER network has still a few disconnected nodes.

Since agents using the RD and BR update rule do not achieve coordination for small values of degree, one might suspect that the network is just not sufficiently connected for these values of the degree, i.e. there are separate components. This is only partially true. In Fig. 3, we can see the structures generated by random regular graph and by ER random graph algorithms. Indeed, for $k=1$ and 2 the topology is trivial and a large (infinite for $k=1$) average path length^23^ can be the underlying feature stopping the system to reach coordination. For $k=3$, however, the network is well connected with one giant component and the system still does not reach the global coordination when using RD or BR. For the UI update rule coordination arrives even for larger values of *k*. Looking at the strategies used by players in Fig. 3 we can see how frozen configuration without coordination can be achieved. There are various types of topological traps where nodes with different strategies are connected, but none of them is willing to change the strategy in the given update rule.

We next consider the question of how the two strategies are distributed in the situations in which full coordination is not reached. Looking at the trajectories in Fig. 1 we can see that there are only few successful strategy updates in such scenario and the value of $\alpha$ remains close to 0.5 until arriving at a frozen state for $k=2$ (also $k=7$ for UI). This suggests that there is not enough time, in the sense of the number of updates, to cluster the different strategies in the network. Therefore, one might expect that they are well mixed as at the end of each simulation. However, an analysis of the density of active links in the final state of the dynamics, presented in Fig. 2, shows a slightly more complex behaviour. When the two strategies are randomly distributed (i.e. well mixed) in a network, the interface density takes the value $\rho =0.5$. When the two strategies are spatially clustered in the network there are only few links connecting them and therefore the interface density takes small values. Looking at the dependence of $\rho$ on *k*, we find that for the replicator dynamics the active link density starts at 0.5 for $k=1$, then drops below 0.2 for $k=2$ and 3 indicating good clustering between strategies, to fall to zero for $k=4$ where full coordination is already obtained. When using the best response update rule the situation is quite different. For $k=1$ there are no active links, $\rho =0$, and hardly any for $k=2$. There is a slight increase of the active link density for $k=3$, to drop to zero again for $k=4$ due to full coordination. Because of the oscillatory level of coordination there are still active links for odd values of $k<10$, but $\rho$ is always smaller than 0.2. In the case of the unconditional imitation we again start at $\rho =0.5$ for $k=1$, before it drops below 0.2 for $k=2$. Subsequently, the active link density grows to obtain its maximum value for $k=7$ and starts decreasing towards zero. These differences in behaviour can be better understood when studying the actual topology of regular graphs for small values of the degree. In Fig. 3 we present frozen configurations for the UI update rule in networks with $k=1,2,3$, and 4. For the smallest degree, $k=1$, the network consists of connected pairs of nodes. Whatever strategies are initially assigned to those pairs they will not change when using RD or UI. In both cases—two nodes using the same strategy and two nodes using different strategies—both nodes in a pair receive the same payoff, therefore no imitation can happen. Hence, the active link density is $\rho =0.5$ for RD and UI. On the other hand, the BR update rule will cause every pair to coordinate, as this is the best possibility for any node, and therefore $\rho =0$. The case of $k=2$ still results in a quite particular structure—it is a chain of nodes (or a 1D lattice). For every update rule the shortest possible cluster of one strategy must contain at least two nodes. The RD and UI separate different strategies relatively well obtaining $\rho <0.2$, however still worse than the BR. For the latter there are almost no active links, i.e. two strategies are perfectly clustered in two (or only few) clusters. From $k=3$ onwards random regular graphs form well connected networks with one giant component and small average path length. This is the largest value of *k* for which the RD and BR update rules do not lead to full coordination. Given the value of the active link density $\rho <0.2$ we can say they both cluster strategies relatively well. For the UI update rule coordination still does not exist for $k=3$, and the number of active links increases with growing degree. This is to be expected—the more links in the network the more connections between nodes playing different strategies. The level of $\rho$ starts to drop only when the coordination begins to settle and we observe that the strategies are well mixed in the network before this point.

**Figure 4:**
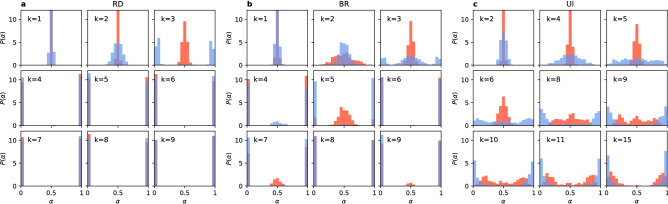
Distribution of the coordination rate $\alpha$ for different values of the average degree *k* and $N=10^3$ for (**a**) the replicator dynamics, (**b**) the best response, and (**c**) the unconditional imitation update rule. Results for random regular networks are presented in red, and equivalent results for random ER networks in blue. Histograms are constructed from a sample of 500 realisations. Note how the distribution changes from unimodal, via trimodal, into bimodal for the UI update rule.

#### Coordination transition

One way of investigating the transition between frozen disorder and global coordination is to look at the probability distribution of the coordination rate $\alpha$. If the distribution is unimodal and centred in the middle at the value of $\alpha =0.5$, we have a disordered state with roughly equal numbers of players using the strategy A and the strategy B. If the distribution is bimodal with two peaks at the boundary values of $\alpha =0$ or 1, all players use the same strategy A or B and coordination was reached. We can see a transition from the first scenario into the second one for all update rules in Fig. 4. The transition point can be identified by the lowest degree for which the distribution is not unimodal (and centred in the middle). The specifics of these transitions, however, differ from one rule to another. For the RD the distribution is unimodal for $k=1,2,3$, becomes trimodal for $k = 4$ (in ER random networks for $k = 3$), and later bimodal for $k \ge 5$. Therefore, the transition point can be defined as the threshold value of the degree $k_c^{RD}=4$. When players use the BR update rule, the distribution becomes bimodal at $k=4$, but is trimodal for $k=5,7,9,11$, i.e. small odd degree values. No such effect of odd degree exists if the network is an ER random network, but also here a trimodal distribution is obtained up to $k=9$. The transition point for the BR is the same as in the RD, $k_c^{BR}=4$, but in the RD beyond this point coordination is always reached, while for the BR there is still a possibility of stopping at a frozen discorded configuration up to $k=11$. While the behavior of the probability distribution of $\alpha$ for the RD and BR update rules is a signature of a first order transition, the transition is less abrupt for the UI update rule. Here the distribution of the coordination rate $\alpha$ is unimodal up to $k=8$, although its variance increases with growing degree of the network. At $k_c^{UI}=9$ the distribution becomes trimodal ($k=6$ for ER random networks), but the side maxima are placed far from the coordinated state—rougly at $\alpha = 0.2$ and 0.8. The trimodal distribution is present up to $k=15$ and the side peaks keep shifting towards boundary values. For $k>15$ the distribution is bimodal, but the peaks are much wider than in other update rules. Additionally, the distribution is not zero between them. This means that the simulation sometimes freezes at a disordered configuration or close to the global coordination, but with a group of agents playing the opposite strategy.

**Figure 5:**
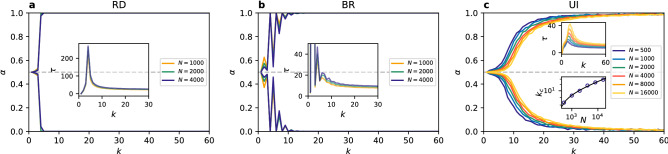
Scaling of the average coordination rate $\alpha$ vs degree of the network *k* for different network sizes using (**a**) the replicator dynamics, (**b**) the best response, and (**c**) the unconditional imitation update rule. The average value is computed from 500 realisations, separately for $\alpha >0.5$ and $\alpha \le 0.5$. Inset plots: scaling of the convergence time $\tau$ vs *k*; additionally in the panel (**c**) scaling of the transition point $k_c$ with the system size *N* with a logarithmic function fit in black. Note, that the behaviour of the system does not depend on *N* for RD and BR, but the transition into coordination shifts towards bigger *k* with growing network for UI.

An important question is what role do the finite size effects play in our results. A larger population could need higher or lower connectivity to obtain the same level of coordination. The answer is given in Fig. 5. From panels (a) and (b) we observe that when players use the RD or BR update rule the resulting level of coordination for a given value of degree is the same for any system size. In other words, even for very large populations we obtain full coordination already for $k=4$. Interestingly, the drop in the coordination rate $\alpha$ for odd values of *k* visible in the BR is also the same for different system sizes. This again suggests that it is an effect of topological traps in regular graphs and not of finite size.

Finite size effects turn out to be quite different when players update their strategies according to the UI update rule. From Fig. 5 we observe that the level of coordination decreases when the population grows at a given fixed connectivity. Or equivalently, the transition from frozen disorder into full coordination is shifted towards higher values of the degree *k*. A proper way of identifying the transition point is by looking at the maximum of the convergence time. In the inset plots of Fig. 5 we see that, when increasing system size, the value of the degree for which the convergence time becomes maximum shift to larger values for the UI update rule, whereas it stays at the same value of *k* for the RD and BR update rules. Additionally, the maximum number of the Monte Carlo time steps necessary to reach a frozen configuration grows with the system size in the case of UI, but stays the same for other update rules. The transition point $k_c$ defined by the maximum convergence time $\tau _{max}$ is equal $k_c^{RD}=4$ for the replicator dynamics, $k_c^{BR}=4$ for the best response, and $k_c^{UI}=9$ for the unconditional imitation for $N=1000$ nodes. Note, that for the BR the convergence time is higher for $k=2$, but we do not take it into account due to the trivial topology. Those threshold values of *k* coincide with the ones discussed above in terms of the changes in the $\alpha$ probability distribution.

The scaling behaviour under the UI update rule raises a question about the minimal connectivity necessary to observe coordination in the thermodynamic limit . In Fig. 5c in the bottom inset plot we present the dependence of the transition point $k_c^{UI}$ on the system size *N*. Two functions can be fitted to these data points—logarithmic function ($R^2=0.997$) and power law ($R^2=0.973$). In the latter case the transition depends on the number of nodes as $k_c^{UI} \sim N^{0.1}$. For both functions the minimum degree required to obtain coordination goes to infinity in the thermodynamic limit. However, $k_c^{UI}/N \rightarrow 0$ when $N \rightarrow \infty$ so that full coordination is achieved in the thermodynamic limit for connectivity much lower than in a complete graph.

Our analysis of the simple PCG already uncovers significant differences among the update rules, including different finite size effects. To achieve global coordination in the population the network must have a minimal connectivity. In random regular graphs this minimum is higher than in ER random networks. Looking at the update rules, players using the RD achieve coordination for the lowest connectivity, or in other words the drive towards global coordination is the strongest in this update rule. When using the BR a slightly higher values of the degree are required for the same level of coordination to appear. Moreover, in random regular graphs the minimal connectivity for the BR update rule is different for odd and even values of the degree. For the UI update rule coordination requires much higher values of the degree *k* and often freezes just before obtaining full coordination (at $\alpha$ close but not equal 0 or 1). System size scaling indicates that even higher connectivity is required for coordination to happen in larger networks, while the transition to coordination for the RD and the BR update rules is size-independent. Interpreting the results one also has to bear in mind that cases of $k=1,2$ produce very particular topologies with large average path lengths, which hinder the coordination. However, for $k \ge 3$ the network is well connected and in principle there is no structural reason why coordination should not emerge. Nevertheless, in many cases it doesn’t.

### General coordination game

After considering the role of local and finite size effects in the simplest case of two equivalent equilibria for coordination, we address in this section the question of equilibrium selection for nonequivalent states of global coordination. We therefore consider the payoff matrix (1) where $R \ne P$. Without loss of generality we can assume that $R>P$ (otherwise we can rename the strategies and shuffle the columns and rows). What defines the outcome of a game are the *greater than* and *smaller than* relations among the payoffs. Therefore we can add/subtract any value from all payoffs, or multiply them by a factor grater than zero, without changing the game. Thus, the payoff matrix (1) can be rewritten as:

3 $$\begin{gathered} \begin{array}{*{20}c} {} & {\qquad {\text{A}}} & {\quad \quad {\text{B}}} \\ \end{array} \;\; \hfill \\ \begin{array}{*{20}c} {\text{A}} \\ {\text{B}} \\ \end{array} \left( {\begin{array}{*{20}c} 1 & {\frac{{S - P}}{{R - P}}} \\ {\frac{{T - P}}{{R - P}}} & 0 \\ \end{array} } \right) \hfill \\ \end{gathered}$$

which, after substituting $S'=\frac{S-P}{R-P}$ and $T'=\frac{T-P}{R-P}$, is equivalent to the matrix:

4 $$\begin{gathered} \begin{array}{*{20}c} {} &\quad \;\;{\text{A}} &\; {\text{B}} \\ \end{array} \;\quad \quad \quad \quad \quad \quad \begin{array}{*{20}c} {} & \quad\; {\text{A}} & \;{\text{B}} \\ \end{array} \hfill \\ \begin{array}{*{20}c} {\text{A}} \\ {\text{B}} \\ \end{array} \left( {\begin{array}{*{20}c} 1 & {S^{\prime}} \\ {T^{\prime}} & 0 \\ \end{array} } \right)\xrightarrow[{{\text{apostrophes}}}]{{{\text{skipping}}}}\begin{array}{*{20}c} {\text{A}} \\ {\text{B}} \\ \end{array} \left( {\begin{array}{*{20}c} 1 & S \\ T & 0 \\ \end{array} } \right) \hfill \\ \end{gathered}$$

**Figure 6:**
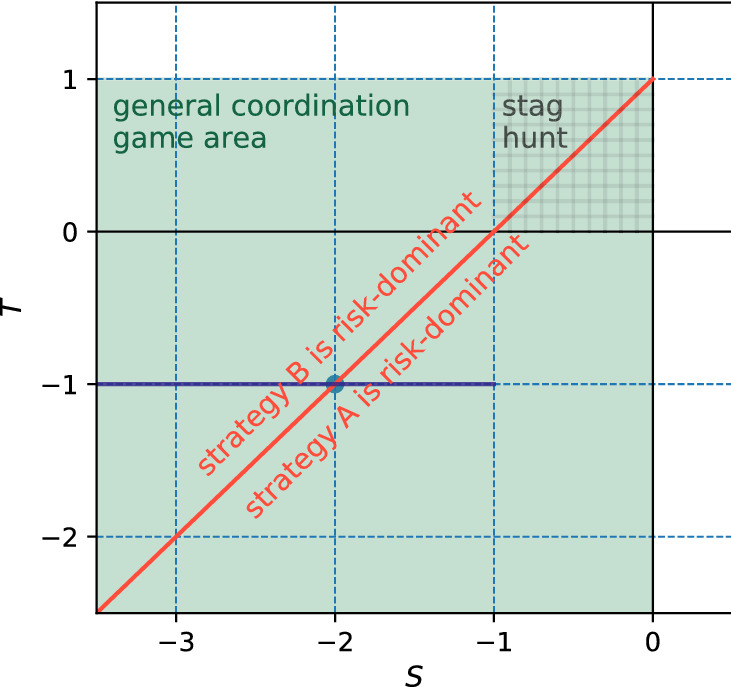
Parameter space of the general coordination game described by the payoff matrix (4), depending on parameters *S* and *T*. The green area shows the region of the general coordination game ($S<0$, $T<1$). The red line represents the line in parameter space at which the risk-dominant strategy changes from A to B (when increasing *T* or decreasing *S*). The purple line represents the parametrisation from the payoff matrix (5) for $b>0$.

From now on we omit the apostrophes and simply refer to parameters *S* and *T*. This payoff matrix can represent many games, including e.g. the prisoner’s dilemma^14,46^ (for $T>1$ and $S<0$). We restrict our analysis to coordination games which correspond to $S<0$ and $T<1$^67,79^. In these games there are pure Nash equilibria at coordinated states, i.e. for both players using the same strategy. We call games that fit in this range of parameters by one name—the General Coordination Game (GCG). In fact, it covers any two-player symmetrical coordination game with different payoffs at coordinated states. For example, the popular game of stag hunt^26,80^ is a special case of the GCG for $0<T<1$ and $-1<S<0$ (the condition $S>-1$ is not always imposed, however conventionally the stag hunt is investigated only in the indicated square area). For $T=-1$ we obtain a game which in a different parametrisation is often studied in literature^42,81^. We shall discuss it in more detail further in the text. Figure 6 illustrates the plane of parameters of the GCG.

**Figure 7:**
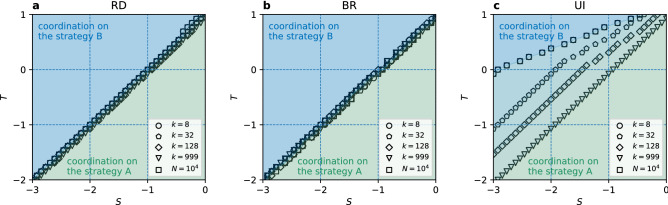
Phase diagram of the general coordination game for (**a**) the replicator dynamics, (**b**) the best response, and (**c**) the unconditional imitation update rule. The green area indicates coordination on the strategy A ($\alpha =1$) and the blue area on the strategy B ($\alpha =0$). Note, that in the panel (**c**) colors blend because of the shift of transition, but for every individual case we obtain coordination below and above the transition line. The transition lines are plotted for $N=10^3$ and different values of *k*, and for $N=10^4$ with $k=8$. Transition lines are obtained from 100 realisations, see Supplementary Figure S1 for the full diagrams.

An important feature of the GCG is that is possesses a payoff-dominant (aka Pareto-optimal) equilibrium and a risk-dominant equilibrium. The strategy A is Pareto-optimal—it provides the largest possible payoff for both players if they coordinate on it. To establish which strategy is risk-dominant we need to compute the average payoffs of strategies A and B, $\Pi _{\mathrm {A}}$ and $\Pi _{\mathrm {B}}$, assuming random and uniform strategy choice in the population. For both strategies having probability 1/2 of being played, from the payoff matrix (4) we obtain $\Pi _{\mathrm {A}} = \frac{S+1}{2}$ and $\Pi _{\mathrm {B}} = \frac{T}{2}$. Therefore, the strategy A will be risk dominant for $T<S+1$ and the strategy B will be risk dominant for $T>S+1$. This calculation provides a theoretical transition line $T=S+1$ between two phases, depending which of the strategies A or B is the risk-dominant strategy (see Fig. 6).

Whether the risk dominance is a sufficient condition for a strategy to prevail in an evolutionary setup is to be examined. It is intuitively clear that when one strategy is both risk-dominant and payoff-dominant at the same time it should be evolutionary favoured. Therefore, we expect to see coordination on the strategy A for $T<S+1$. The question is what happens when there is a choice between a risk-dominant strategy and a payoff-dominant one. We explore in the following paragraphs the effect of update rule, local effects and finite size in equilibrium selection.

We present phase diagrams obtained from our numerical simulations for the three update rules in Fig. 7. For the replicator dynamics and the best response update rules there is a transition in the equilibrium selection at the line $T=S+1$. For $T<S+1$ the strategy A, which is there both payoff-dominant and risk-dominant, is selected, but for $T>S+1$ the risk-dominant strategy B is selected. Importantly, the transition line from A to B selection is independent of the degree or size of the network. These findings are also consistent with analytical calculations based on the replicator equation for the RD and mean field approach for the BR (see “Methods” for details). Interestingly, when players use the unconditional imitation the transition is shifted towards larger values of *T* (and smaller *S*) for sparser networks. The transition line moves to $T=S+1$ with growing degree of the network and finally reaches this line for a complete graph. Note, that for the stag hunt area and small values of *k* our results are consistent with Roca et al.^46^.

The important consequence of local effects is that players can still coordinate on the Pareto-optimal strategy A even when this strategy is far from being risk-dominant. However, this only happens for the UI update rule and small enough connectivity in the population. This is in a sense opposite to the results in the PCG. There, the optimal outcome, i.e. any coordination, could be achieved only above a threshold value of the degree. Here, for a given range of the parameters *S* and *T* the optimal outcome, i.e. coordination on the Pareto-optimal strategy, can be achieved only for networks with small enough connectivity. It is important to note that a previous most explicit result on the importance of local effects was given for rings (circular city)^56^. The behaviour there was different—for sparse networks a larger connectivity was required to coordinate on the Pareto-optimal strategy (for densely connected networks the risk-dominant equilibrium was indirectly suggested). Including more complex structure without noise in strategy imitation, as we show, changes this relation. One might imagine that our results could change when analysing networks with the degree smaller than 8. In such sparse networks we observe coordination on the Pareto-optimal strategy or no coordination. However, the risk-dominant equilibrium is the only possibility for very small values of the parameter *S* (see Fig. 10, Supplementary Figures S2, S3 for results on very sparse networks).

**Figure 8:**
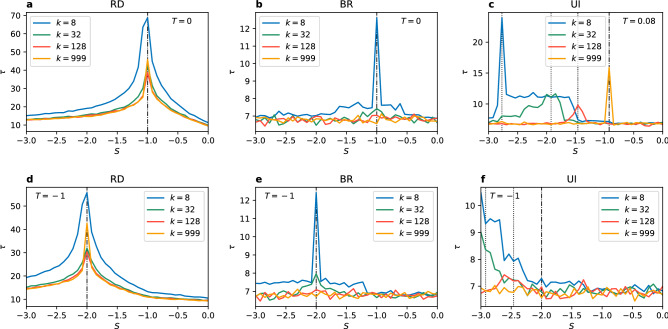
Convergence time $\tau$, counted in Monte Carlo steps, in the general coordination game vs parameter *S* for $N=10^3$ and different values of the degree *k*. The update rule is (**a**,**d**) the replicator dynamics, (**b**,**e**) the best response, and (**c**,**f**) the unconditional imitation. The upper row (**a**,**b**) presents results for $T=0$, except (**c**) where $T=0.08$. The bottom row (**d**–**f**) presents results for $T=-1$. Vertical dashed lines mark the value $S_c$ at which the risk-dominant strategy changes from A to B. Dotted lines show the transition point from Fig. 7 (dashed-dotted indicate both). All values are averaged over 100 realisations.

We next consider the average convergence time $\tau$ to the selected equilibrium. Results for two different values of *T* are shown in Fig. 8. As previously, RD is the update rule least affected by local effects (changes in the value of the degree). A maximum convergence time is always found at the transition point of equilibrium selection. Additionally, the time to reach a frozen configuration is similar for all values of *k*, except sparse networks ($k=8$) where it becomes bigger. When using the BR update rule converge times are in general slightly shorter. For smaller degree ($k=8$) the transition point of equilibrium selection is firmly marked by a narrowly peaked maximum value of $\tau$. For $k=32$ there is very small increase in the convergence time and for larger values of degree the transition is not identified at all. In the case of the UI update rule the transition in equilibrium selection does not coincide with the change of risk-dominant strategy and the equilibrium selection transition only manifests itself as a maximum in the convergence time for $T>0$. In the panel (c) of Fig. 8 the maximum of $\tau$ is obtained at a different point for each value of *k*, because the equilibrium selection transition moves with changing degree. Every peak corresponds to the transition for the given *k*. For $T \le 0$, however, the maximum convergence time is not associated with the transition. Note, that comparing this behaviour with our results for the PCG, one must remember that in the PCG the maximum of the convergence time was associated with a transition from a frozen disorder to coordination, while in the GCG the transition is only a change of the selected equilibrium of full coordination.

#### Local effects and size effects in GCG

A particular coordination game often studied in the literature is the one described by the following payoff matrix^42,43,56,81^:

5 $$\begin{gathered} \begin{array}{*{20}c} {} & \qquad {\text{B}} & \;{\text{A}} \\ \end{array} \hfill \\ \begin{array}{*{20}c} {\text{B}} \\ {\text{A}} \\ \end{array} \left( {\begin{array}{*{20}c} 1 & 0 \\ { - b} & 2 \\ \end{array} } \right) \hfill \\ \end{gathered}$$

where usually a restriction $b>0$ is imposed, although it is still a coordination game up to $b>-1$. Note, that the strategy symbols changed their positions. In a typical notation the upper left strategy is Pareto-optimal, i.e. it is the strategy that gives the largest payoff. We denoted it before as the strategy A. Therefore, to stay consistent we change the denotation to maintain (A, A) the Pareto-optimal configuration.

The game described by the payoff matrix (5) is fully equivalent to the GCG with $T=-1$. To see this, it is enough to interchange the positions of strategies A and B and subtract 1 from every entry to obtain:

6 $$\begin{gathered} \begin{array}{*{20}c} {} & \quad\;\,{\text{A}} &\; \,{\text{B}} \\ \end{array} \;\quad \quad \quad \quad \quad \quad \begin{array}{*{20}c} {} & \qquad{\text{A}} &\qquad {\text{B}} \\ \end{array} \hfill \\ \begin{array}{*{20}c} {\text{A}} \\ {\text{B}} \\ \end{array} \left( {\begin{array}{*{20}c} 2 & { - b} \\ 0 & 1 \\ \end{array} } \right)\xrightarrow{{{\text{subtracting}}\;1}}\begin{array}{*{20}c} {\text{A}} \\ {\text{B}} \\ \end{array} \left( {\begin{array}{*{20}c} 1 & { - b - 1} \\ { - 1} & 0 \\ \end{array} } \right) \hfill \\ \end{gathered}$$

In this notation it is clear that the game described in the literature by the payoff matrix (5) is equivalent to the GCG described by the payoff matrix (4) for $T=-1$ and $S=-b-1$. In this parametrisation the change of risk-dominant strategy from A to B lays at $S=-2$ ($b=1$). For $S>-2$ ($b<1$) the strategy A is risk-dominant and for $S<-2$ ($b>1$) the strategy B is risk-dominant. For the RD or BR update rules the transition in equilibrium selection obtained in numerical simulations presented in Fig. 7 occurs at this same point $S_c=-2$ ($b_c=1$) (also in ER random networks, not presented due to identical character). For this reason, here we focus on transition for the unconditional imitation update rule. In order to study how does equilibrium selection depend on the degree *k*, we have to fix the payoff matrix. We consider the case with $T=-1$ that was already studied in the literature (under parametrisation with *b*). Then, we analyse local effects and size effects in coordination and equilibrium selection for different values of the parameter *S*.

**Figure 9:**
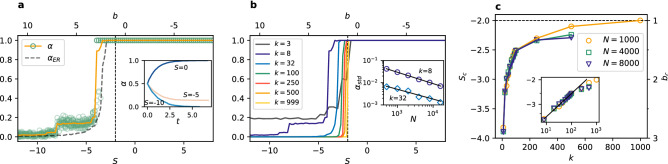
(**a**) Coordination rate $\alpha$ vs parameter *S* (and *b*) for $N=1000$ and $k=8$. Each green circle represents one of 100 realisations for each value of *S* and the average value is plotted with a solid line. Results are compared to the ER random network ($\alpha _{ER}$). Inset plot: time evolution of $\alpha$ in representative realizations for 3 values of *S*. (**b**) The average value of $\alpha$ vs. parameter *S* (and *b*) for different values of the degree *k* and $N=1000$. Inset plot: scaling of the standard deviation $\alpha _{std}$ with the network size for $S=-6$ and $k=8,k=32$; black lines show the best power law fit. (**c**) The point of transition in equilibrium selection $S_c$ (and $b_c$) vs degree *k* for different network sizes. Inset plot: the same results on a log-log scale with a power law fit up to $k=250$. In all plots vertical (**a**,**b**) and horizontal (**c**) dashed lines mark the point of change in risk-dominant strategy $S=-2$ ($b=1$). The update rule is unconditional imitation.

In Fig. 9a we present the dependence of coordination rate $\alpha$ on the parameter *S* (and *b*). We can see that the transition in equilibrium selection from $\alpha =0$ to $\alpha =1$ is shifted towards lower values of *S* (or larger values of *b*) in comparison to the values at which risk-dominant strategy changes. Interestingly, all realisations lay very close to the average value of $\alpha$. Therefore, the coordination is well predefined by the parameter *S* (with other parameters fixed). We can see that in ER random graphs the transition in equilibrium selection is slightly closer to the $S=-2$ ($b=1$) point and also the increase of coordination rate is smoother. The inset of panel (a) shows representative trajectories, which quickly converge into a frozen configuration for different parameter values. In Fig. 9b we show the average coordination rate $\alpha$ for different degrees of the network. The transition point $S_c$ of equilibrium selection from B-coordination to A-coordination shifts towards the value $S=-2$ with growing connectivity to finally coincide with this point of change in the risk-dominant strategy for a complete graph. Additionally, the coordination rate changes directly from $\alpha =0$ to $\alpha =1$ for higher degree values, without the intermediate plateau visible for $k=8$ (see Supplementary Figures S2, S3 for results on very sparse networks). These results are robust regardless of the network size, as the standard deviation $\alpha _{std}$ decreases with growing *N* (see the inset plot of Fig. 9b). In order to investigate further the dependence of the transition point $S_c$ (and $b_c$) on the network’s degree we plot it for different network sizes in Fig. 9c. Up to $k \approx 250$ the transition point follows a power law $S_c(k) \sim k^{-0.17}$ ($R^2>0.99$) for every size of the network. Then, the lines separate as each of them has the upper limit of $S_c=-2$ which is obtained for a complete graph, i.e. for $k=N-1$ which depends on the number of nodes. Note, that the found dependence $S_c(k)$ can be reversed to obtain the critical value of the connectivity $k_c(S) \sim S^{5.88}$ above which the system coordinates at the risk-dominant equilibrium and below at the payoff-dominant one.

**Figure 10:**
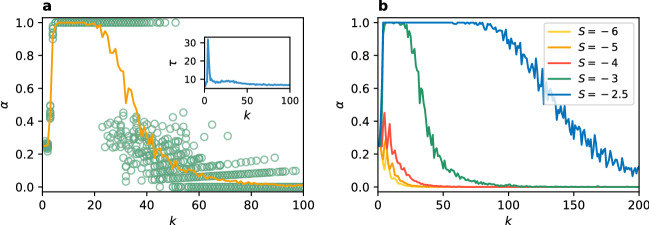
Coordination rate $\alpha$ vs degree *k* for $N=1000$ and $T=-1$. (**a**) Results for $S=-3$, the green circles represent one of 100 realisations for each degree value, the orange line shows the average value. Inset plot: convergence time $\tau$ vs *k*. (**b**) The average result for $S=-2.5$, $-3$, $-4$, $-5$, and $-6$ (over 100 realisations). The update rule is unconditional imitation.

Finally, to study the degree dependence for particular values of the payoff matrix we set both parameters *T* and *S* to specific values and run simulation for various connectivities, as presented in Fig. 10. In the panel (a) for $T=-1$ and $S=-3$ we can see that for very sparse networks ($k=2$ and 3) coordination can not be obtained and the system freezes in a disordered configuration (see Supplementary Figure S3 for a close-up on very sparse networks). Then, for a range of degrees from 4 to approximately 20 the system fully coordinates exclusively on the strategy A, i.e. in the Pareto-optimal equilibrium. Increasing *k* further a discontinuous transition with hysteresis follows. Up to approximately $k=55$ the players can either fully coordinate in the payoff-dominant equilibrium or end up in a frozen configuration with $\alpha \in [0, 0.5]$—a disordered state or a risk-dominant equilibrium. For even bigger connectivity only the frozen disorder or risk-dominant equilibrium are possible, to finally around $k=100$ coordinate at the risk-dominant equilibrium in every realisation.

In Fig. 10b, we present the average coordination rate for a larger range of connectivity and several values of the parameter *S*. We only show results for $S<-2$, since for $S>-2$ the same strategy A is both payoff- and risk-dominant. Therefore, the game lacks the competition between those two factors and the strategy A is always chosen in the full coordination state. As visible in the figure, the phase of Pareto-optimal equilibrium is present only for larger values of the parameter *S*. For $S=-4$ and smaller the system never coordinates on the payoff-dominant strategy. Instead we observe a transition from no coordination directly into the risk-dominant equilibrium. This is consistent with the results from Fig. 9b where we could see that the Pareto-optimal coordination could be obtained only for a small range of *S* below $-2$ for every value of the degree. One can say that the Pareto-optimal equilibrium is fragile, it requires fine tuning of the parameters. For $T=-1$ the parameter *S* must be bigger than $-4$ and the network can not be very sparse nor too densely connected.

## Discussion

The three update rules considered in this paper assume that a rational agent aims at increasing its payoff. Either by directly computing the payoff and choosing the larger one like in the BR update rule (therefore this rule requires players’ knowledge about the payoff matrix), or by imitating a more successful neighbour with a larger payoff like in the RD and UI updates rules. Note, that the UI and BR updates rules are equivalent in a fully connected network. However, the outcome can be very different between those three update rules in coordination games in random networks, even for the simplest pure coordination game. A simple one-round two-player game is fully described by its payoff matrix, but an evolutionary game is defined by the update rule as much as by the payoff matrix. We focused on the local effects and finite size effects which are crucial in networked populations. It turns out that these effects are much more important for the UI update rule.

In the pure coordination game, we addressed the question of when the system reaches full coordination and when it is trapped in a frozen disordered state of coexistence of the two equivalent strategies. We found a transition from a disordered state to a state of full coordination for a critical value $k_c$ of the degree: global coordination requires a minimum connectivity of the network. The critical value is well identified by a maximal value of the convergence time. The transition is discontinuous or first-order like for the RD and BR update rules, and continuous for the UI update rule. The value of $k_c$ is different for the different update rules, but it is system size independent for the RD and BR and system size dependent for the UI. Still, also for the UI update rule the transition remains well defined in the limit of an infinite network (thermodynamic limit), so that global coordination is obtained for a connectivity lower than in a complete graph.

For the general coordination game, we addressed the question of equilibrium selection: coordination in either a payoff-dominant or risk-dominant equilibrium. A mean field approximation for the BR or UI and the replicator equation for the RD update rule predicts that the risk-dominant equilibrium is always selected regardless if it is Pareto-optimal or not. This prediction implies that there is a transition from selecting strategy A to selecting strategy B at the point at which the risk-dominant strategy changes from A to B. This prediction is corroborated by our numerical simulations for RD and BR update rules independently of local effects (connectivity *k*) and system size. However, for the UI update rule we find that it is possible to select the Pareto-optimal strategy A even when it is not the risk-dominant one. This is a consequence of local effects—such selection is possible only when the network is not too densely connected. Our detailed analysis identifies a critical value of the parameters for this transition in equilibrium selection that depends on the network degree, or alternatively a critical value of the degree such that for lower values of the degree the Pareto-optimal strategy is selected. For very sparse networks, however, we do not obtain coordination and the system freezes in a disordered state. Therefore, we have two possible scenarios of degree dependence based on the parameters *T* and *S*. In the first one we have no coordination for very sparse networks, increasing the degree we obtain coordination on the Pareto-optimal strategy, and increasing the degree even further we obtain coordination on the risk-dominant strategy. In the second scenario the Pareto-optimal equilibrium does not exist and the system goes from no coordination directly into the risk-dominant equilibrium when increasing the connectivity. In summary, it is a combination of local effects and update rule that makes coordination on the payoff-dominant strategy A when it is not risk-dominant possible. We note that our results are different from those obtained in a circular topology^56^ where higher connectivity (but far from the complete graph limit) favours selection of the payoff-dominant strategy. This difference could be explained by a different network structure and no random mutations in our model. However, a different study of the circular city model obtained results qualitatively consistent with ours for non-sparse networks. Namely, when increasing the interaction group the probability of reaching the Pareto-optimal equilibrium was decreasing^65^. Nevertheless, this work considered more complex structure with a temporal network of interactions and a separate information network.

Our results are general and cover many applications of evolutionary coordination games. We shed light on the conditions required to obtain any coordination in networked populations. Additionally, we show when the Pareto-optimal strategy is chosen for the coordinated state. Our work is relevant in human and animal cooperation issues, biological, social and economical sciences, and other fields applying evolutionary game theory. The payoff matrices we analysed are comprehensive and cover all symmetrical coordination games with either equivalent equilibria or with one strategy being risk-dominant and one payoff-dominant (it can be the same strategy). Future work could cover asymmetrical coordination games^82,83^ such as battle of sexes and include noise or error in strategy selection, which could further enhance coordination^47,84^.

## Methods

### Mean-field description of BR and UI

To study the dynamics of the system we consider the coordination rate $\alpha$ accounting for the number of nodes playing the strategy A (divided by the network size *N* for normalisation). The probability of finding neighbours having a particular state is not necessarily uniform, however the simulations suggest that strategies are well mixed in most of the scenarios. Therefore, we can make the mean field assumption that $\alpha k$ neighbours of a randomly chosen node will play the strategy A and $(1-\alpha ) k$ the strategy B. Accordingly, the expected payoffs from the strategies in the general coordination game described by the payoff matrix (4) will be:

7 $$\begin{aligned} \Pi _{\mathrm {A}} &= {\alpha } k \cdot 1 + (1-\alpha ) k \cdot S = k(\alpha -\alpha S +S) ,\\\Pi _{\mathrm {B}} &= {\alpha } k \cdot T + (1-\alpha ) k \cdot 0 = {\alpha } k T. \end{aligned}$$

When using the best response update rule every node deliberately chooses the strategy that will result in the highest payoff, hence the condition for the strategy A to be chosen is simply $\Pi _{\mathrm {A}} > \Pi _{\mathrm {B}}$, which leads to the condition:

8 $$\begin{aligned} k(\alpha -\alpha S +S)> {\alpha } k T ~\implies ~ \alpha + \frac{S}{1-S-T} > 0, \end{aligned}$$

with the constraints of the coordination game $S<0$ and $T<1$. Finally, the rate of adoption of a given strategy is proportional to the fraction of nodes using the opposite strategy leading to a time dependence described by:

9 $$\begin{aligned} \frac{\partial {\alpha }}{\partial t} = \frac{k}{N} \left[ \theta \left( {\alpha } + \frac{S}{1-S-T} \right) - {\alpha } \right] , \end{aligned}$$

where $\theta$ is the Heaviside step function. If we take $\alpha = 0.5$, which is the initial value used in the simulations, in the equation above we can see which strategy is selected. More precisely, from the argument of the Heaviside function we obtain the inequality (8), but with $\alpha = 0.5$, from which we obtain a condition for strategies to be evolutionary chosen: $T<S+1$ for the strategy A and the opposite for the strategy B. Note, that this is the same condition as for strategies to be risk-dominant, so that the mean field approximation predicts that the risk-dominant equilibrium is selected. The same analysis can be applied to unconditional imitation with large average degree. The active node will imitate the most successful neighbour in the network, which in a complete graph is the best choice in terms of the future payoff, because all nodes have the same neighbourhood. Therefore, effectively the active node will choose the most lucrative strategy as in the best response. This, however, might not be true in sparse networks.

### Replicator equation for RD

A population of players using replicator dynamics is described by the general replicator equation:

10 $$\begin{aligned} \dot{x}_i = x_i ( {\hat{e}}_i \cdot M \vec {x} - \vec {x} \cdot M \vec {x} ) , \end{aligned}$$

where *M* is the payoff matrix and $\vec {x} = [x_A , x_B]$ accounts for the fraction $x_A$ of individuals using the strategy A and $x_B$ using the strategy B, i.e. $x_A=\alpha$ and $x_B=1-\alpha$. Therefore, ${\hat{e}}_i \cdot M \vec {x}$ is the average payoff of individuals playing strategy *i* and $\vec {x} \cdot M \vec {x}$ is the average payoff in the whole population. Using the constraint $x_B = 1 - x_A$ and the exact form of the payoff matrix *M* in the general coordination game (4) we obtain the equation for dynamics of the coordination rate $\alpha =x_A$:

11 $$\begin{aligned} \frac{\partial {\alpha }}{\partial t} = (S+T-1) \alpha ^3 + (1-2S-T) \alpha ^2 +S \alpha . \end{aligned}$$

It has three stationary solutions at $\alpha ^* \in \{ 0, \frac{S}{S+T-1}, 1 \}$. However, the solution with no full coordination $\alpha ^*= \frac{S}{S+T-1}$ is linearly unstable because the inequality $S+T-1 <0$ is always satisfied for a coordination game ($S<0$ and $T<1$). The change of sign of $\frac{\partial {\alpha }}{\partial t}$ in (11) also takes place at $\alpha ^*= \frac{S}{S+T-1}$. Therefore, in order to reach the coordination at the strategy A ($\alpha =1$) the condition:

12 $$\begin{aligned} \alpha > \frac{S}{S+T-1} \end{aligned}$$

must be fulfilled, which is equivalent to the condition (8) obtained in the mean-field approach for the BR. Taking into account the initial condition of $\alpha =0.5$ we obtain the inequality $T<S+1$ for the strategy A to be evolutionary chosen. Therefore, the RD equation also predicts that that the risk-dominant equilibrium is selected.

## Supplementary Information

Supplementary Information.
