# Supplementary material for: Coordination and equilibrium selection in games: the role of local effects
Source: Sci Rep. 2022 Mar 1;12:3373. doi: 10.1038/s41598-022-07195-3 (PMC8888577; doi:10.1038/s41598-022-07195-3)
Supplement: Supplementary file 1 — Supplementary Information. [file 41598_2022_7195_MOESM1_ESM.pdf]

Supplementary material for
Coordination and equilibrium selection in games: the role of local effects

Tomasz Raducha
tomasz@ifisc.uib-csic.es
Instituto de Física Interdisciplinar y Sistemas Complejos IFISC (CSIC-UIB), Palma, Spain
Institute of Experimental Physics, Faculty of Physics, University of Warsaw, Warsaw, Poland

Maxi San Miguel
Instituto de Física Interdisciplinar y Sistemas Complejos IFISC (CSIC-UIB), Palma, Spain

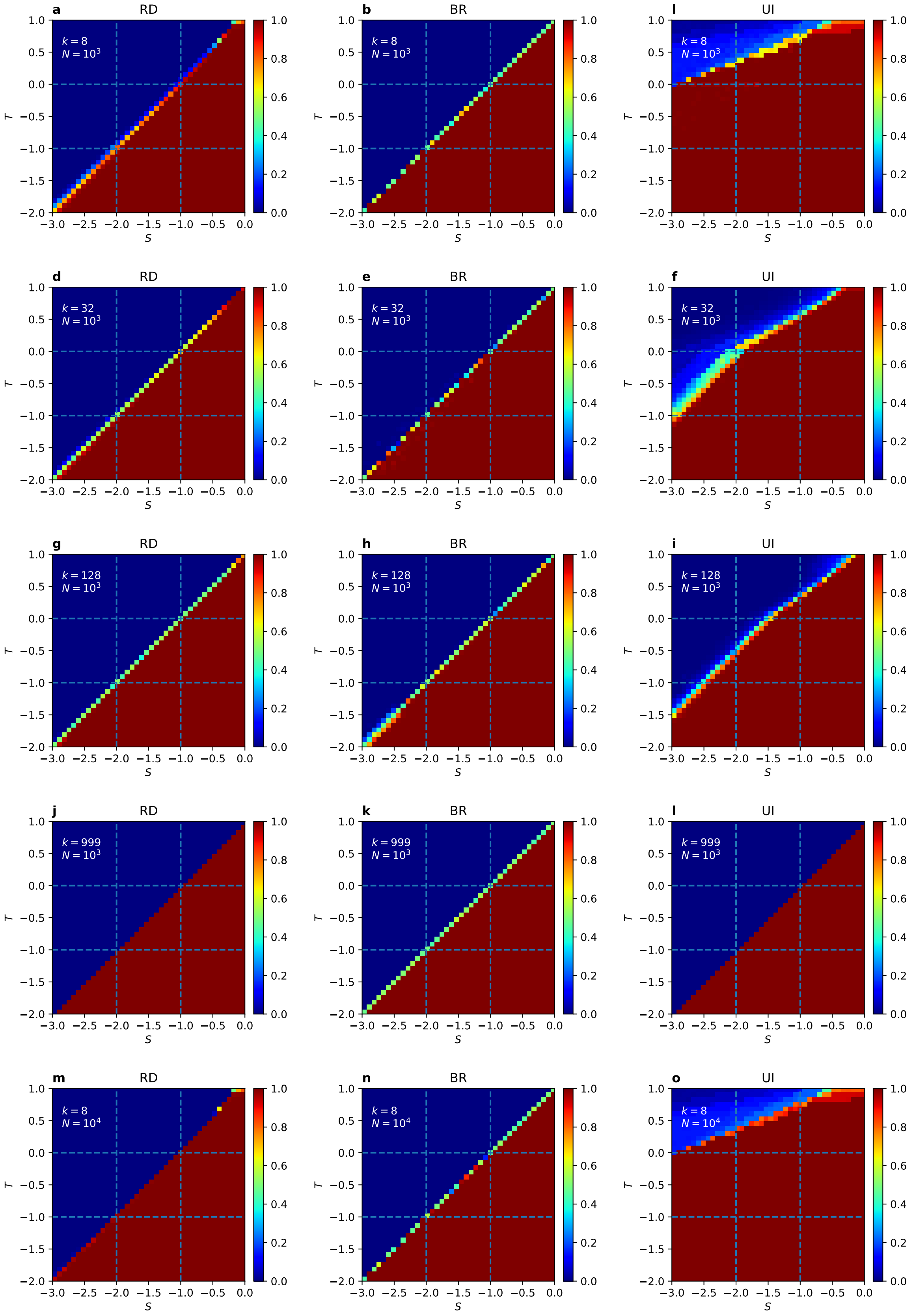

Supplementary Figure S1: Phase diagram of the general coordination game – coordination rate α vs parameters S and T for (a, d, g, j, m) the replicator dynamics, (b, e, h, k, n) the best response, and (c, f, i, l, o) the unconditional imitation update rule. The diagrams are computed for $N = 10^3$ and different values of degree k: (a, b, c) $k = 8$, (d, e, f) $k = 32$, (g, h, i) $k = 128$, and (j, k, l) $k = 999$ (complete graph), except the bottom row (m, n, o) which presents diagrams for $N = 10^4$ and $k = 8$. Results are obtained from 100 realisations.

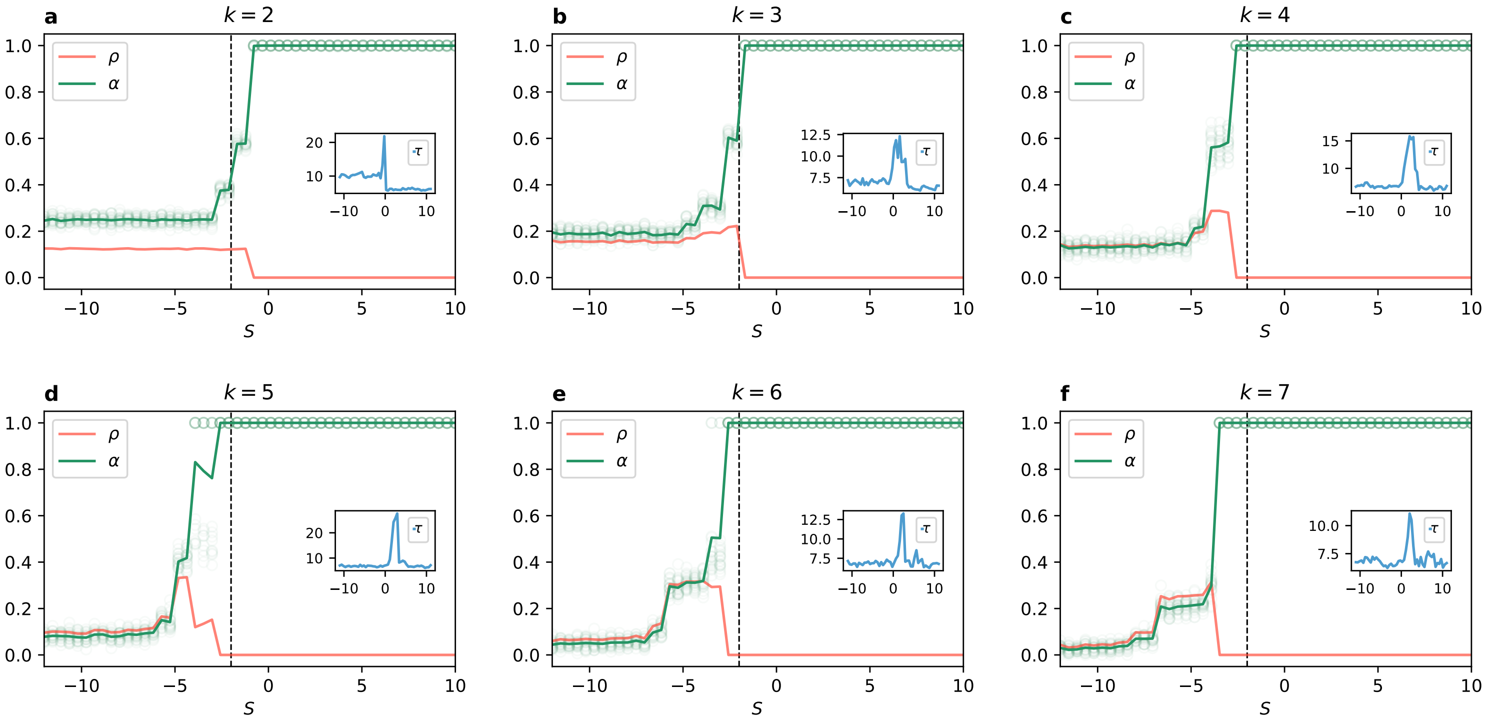

Supplementary Figure S2: Coordination rate α and interface density ρ vs parameter S for $T = -1$, $N = 1000$, and different values of the degree k. Each green circle represents one of 20 realisations for each value of S and the average value is plotted with a solid line. Inset plot: converge time τ (in MC setps) vs S.

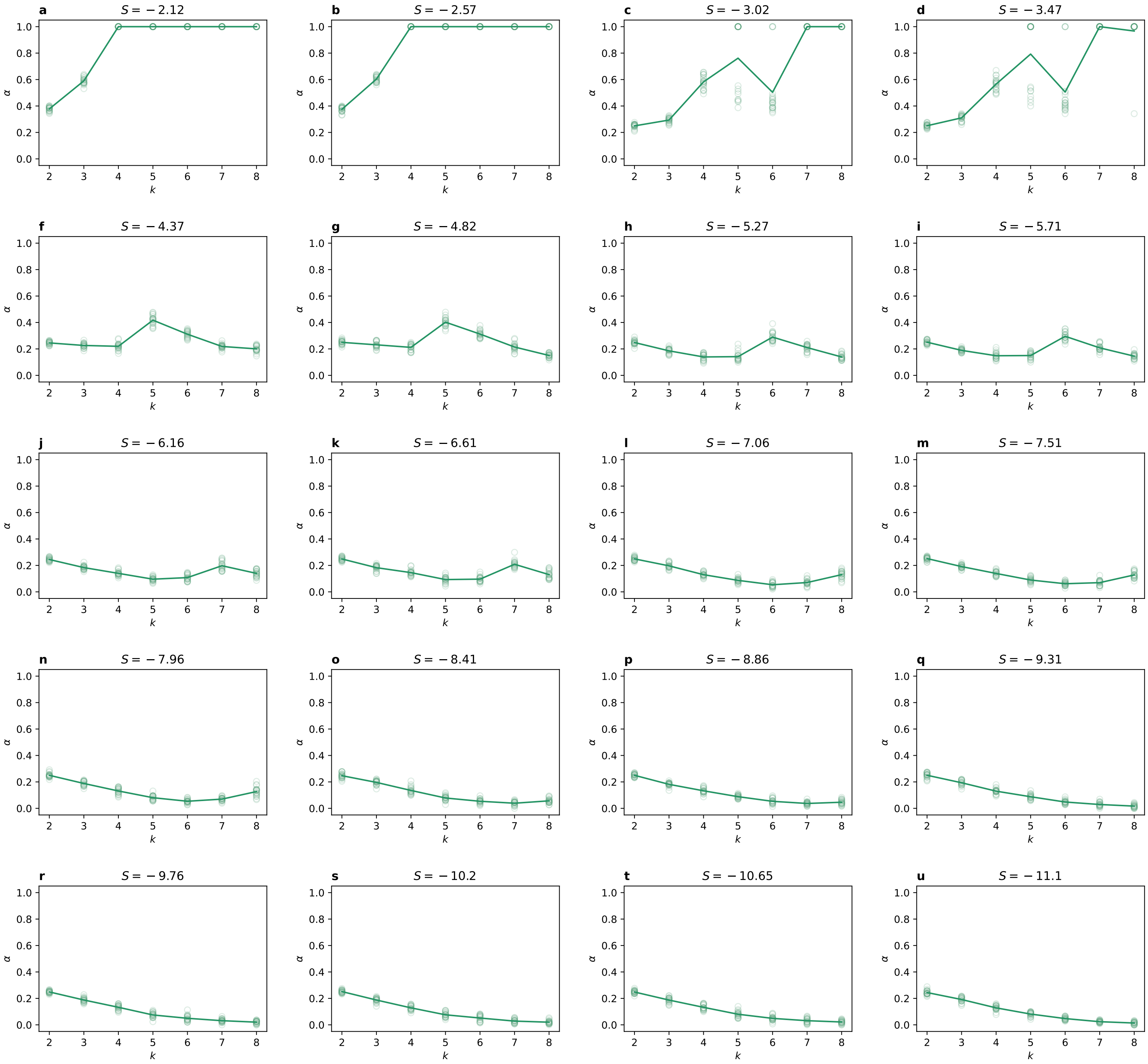

Supplementary Figure S3: Coordination rate α vs degree k for $T = -1$, $N = 1000$, and different values of the parameter S. Each green circle represents one of 20 realisations for each value of k and the average value is plotted with a solid line.
